# A Digital Dietary Assessment Tool for a Multicultural Population (Intake24 Malaysia): Protocol for a Development and Validation Study

**DOI:** 10.2196/90106

**Published:** 2026-08-04

**Authors:** Nik Nur Izzati Nik Mohd Fakhruddin, Shaleen Tho Rajasegaram, Nurul Natasha Osman, Emma Foster, Choon Ming Ng, Nur Suraiya Abu Hassan Shaari, Tracy McCaffrey, Christopher Prawira, Patrick Olivier, Jessica Watterson, Amutha Ramadas

**Affiliations:** 1 Jeffrey Cheah School of Medicine and Health Sciences, Monash University, Jalan Lagoon Selatan, Subang Jaya, Selangor, 47500, Malaysia, 60 5146000; 2 Action Lab, Department of Human-Centred Computing, Faculty of Information Technology, Monash University, Melbourne, Victoria, Australia; 3 Centre for Holistic Initiatives for Learning and Development (CHILD), Yong Loo Lin School of Medicine, National University of Singapore, Singapore, Singapore; 4 Faculty of Health Sciences, Centre for Dietetics Studies, Universiti Teknologi MARA, Puncak Alam, Selangor, Malaysia; 5 Department of Nutrition, Dietetics and Food, Monash University, Notting Hill, Victoria, Australia; 6 Institute for Health Transformation, School of Health and Social Development, Deakin University, Burwood, Victoria, Australia

**Keywords:** nutrition assessment, ethnicity, Malaysia, dietary intake, surveys and questionnaires

## Abstract

**Background:**

Malaysia is a multicultural country with the main ethnic groups being Malays, Chinese, and Indians. This diversity creates a rich food culture with distinct dishes, cooking styles, and portion sizes, making dietary assessment challenging. Intake24 is a web-based 24-hour dietary recall (24hDR) system that automates data collection, reduces recall bias, and saves time. It supports self-reporting of the previous day’s food and beverage intake using structured prompts, searchable food lists, portion-size images, and linked food composition data. As it was originally developed in the United Kingdom, adaptation for Malaysia is needed to address differences in language, food culture, mixed dishes, portion sizes, terminology, and local food composition data.

**Objective:**

This paper aims to describe a protocol for the development and relative validation of Intake24 Malaysia (Intake24-MY) for the Malaysian population.

**Methods:**

This paper describes 2 phases in adapting Intake24-MY: (1) the development process and (2) the validation study. Phase 1 consists of the following components: (1a) system translation, which will involve machine translation tools and bilingual translators; (1b) food list development, which includes Malaysian and globally familiar foods; (1c) 4 options for portion-size estimation and a photograph validation study; (1d) food composition data and recipe calculation; (1e) small-scale testing, which will involve 10 adults identifying the foods and technical issues, with pilot testing conducted among 100 adults to compare 2 days of dietary intake using Intake24-MY against an interviewer-led 24hDR; and (1f) user guide development. Phase 2 consists of the following components: (2a) a single-meal validation study that will be conducted among 100 adults, comparing Intake24-MY with observed intake and (2b) a cross-sectional study that will be conducted among 482 Malaysian adults, comparing 4 days of dietary intake using Intake24-MY against an interviewer-led 24hDR. A structured questionnaire will be used to assess the feedback on the usability of Intake24-MY. The Bland-Altman method will be used to determine the agreement between these methods.

**Results:**

The study was funded in December 2023, with data collection starting in June 2024 and continuing. Data analysis is set to begin in August 2026, with results expected by 2027. This study has received approval from the Monash University Human Research Ethics Committee (MUHREC ID 41337).

**Conclusions:**

Intake24-MY is being developed as a multilingual digital dietary assessment tool for Malaysia. The planned validation study will determine its agreement with interviewer-led 24hDRs and assess its usability among Malaysian adults. If shown to be valid and acceptable, Intake24-MY can support more efficient dietary data collection in Malaysia’s multicultural population.

## Introduction

A suboptimal diet is a significant risk factor for noncommunicable diseases, including type 2 diabetes, hypertension, and cardiovascular disease, and contributes to a disproportionately high disease burden in Malaysia [1,2]. Although diet is a key modifiable risk factor, it is often underassessed in research, particularly in low- and middle-income countries (LMICs), where the lack of appropriate dietary assessment tools poses a significant barrier to large-scale dietary data collection [3,4].

Direct dietary assessment methods, which collect information directly from participants, are commonly used to examine the relationship between diet and disease [5]. Table 1 summarizes the 6 primary types of direct dietary assessment methods [5,6], among which the weighed food record is considered the gold standard [7]. However, this method imposes a significant burden on participants, who must weigh all foods before and after consumption [8,9]. Consequently, methods such as food records, food frequency questionnaires, and 24-hour dietary recalls (24hDRs) are more commonly used in large-scale epidemiological research [10,11]. Each method has its strengths and limitations, but 24hDRs are often regarded as a practical compromise because they can capture detailed dietary intake data without overburdening participants. Nonetheless, this method remains resource-intensive, requiring skilled interviewers, which contributes to high costs and time demands [7,11,12]. Recent technological advancements, however, have helped address some of these challenges, making large-scale dietary data collection more feasible [13].

**Table 1.** Primary dietary assessment methods.

Categories and tools	Methods	Collected date
Methods of real-time recording		
Duplicate diet approach	Collection of duplicate diet samples and direct analysis	Actual intake information throughout a specific period
Food consumption records	Objective observation by trained staff at the household level	Actual intake information throughout a specific period
Dietary record	Subjective measure using open-ended, self-administered questionnaires	Actual intake information throughout a specific period
Methods of recall		
Single or multiple daily recalls	Subjective measure using open-ended questionnaires administered by a trained interviewer	Actual intake information over the previous 24 hours
Dietary histories	Subjective measures using open-ended and closed-ended questionnaires administered by a trained interviewer	Usual intake estimates over a relatively long period
Food frequency questionnaire	Subjective measure using a predefined, self-administered, or interviewer-administered format	Usual intake estimates over a relatively long period (eg, 6 months or 1 year)

Digitalization of dietary assessment in LMICs offers significant benefits, particularly in multicultural settings such as Malaysia, by enhancing data accuracy, efficiency, and inclusivity. Traditional paper-based methods are resource-intensive and prone to errors. In contrast, digital tools enable real-time data collection through automated prompts, precoded multilingual food lists, and culturally relevant portion-size images, thereby minimizing recall bias and transcription errors [5,14]. Advances in technology have led to the development of various digital dietary assessment methods, ranging from smartphone-based image capture to internet-delivered adaptations of traditional approaches [15]. These innovations lower costs, reduce participant and researcher workloads, and improve data consistency through automated coding.

One such digital tool is Intake24 [16], a web-based 24hDR system initially developed and validated [17] for use in the United Kingdom and implemented in the National Diet and Nutrition Surveys [18]. Since its inception, Intake24 has been adopted in various regions, including the United Arab Emirates, Portugal, Denmark, South Asia, and Australia. Intake24 is designed to allow users to self-report all foods and beverages consumed during the previous day. Unlike traditional interviewer-led recalls, Intake24 guides users through a structured multiple-pass recall process, supported by searchable food lists, portion-size images, automated prompts, and linkage to food composition data. These features can reduce researcher burden, improve standardization, and enable efficient dietary data collection at scale. However, because Intake24 was originally developed in the United Kingdom, its direct application to the Malaysian population is limited by differences in language, food culture, mixed-dish composition, food terminology, portion-size norms, and the availability of food composition data. Malaysia’s multicultural dietary landscape includes Malay, Chinese, Indian, Indigenous, and regional food practices, with many foods consumed as composite dishes, shared dishes, or meals with multiple accompaniments. Therefore, adaptation of Intake24 for Malaysia requires more than direct translation; it involves cultural and linguistic localization of the food database, portion-size estimation methods, food descriptors, associated food prompts, and nutrient database linkage. Each recall takes approximately 20 minutes to complete, after which the nutritional composition of the reported intake is immediately available for download. Intake24 also includes features such as prompts for commonly paired foods, contextual help messages, a demonstration video, and optional personalized dietary feedback.

Malaysia is a multicultural country comprising 70.3% Bumiputera (58.1% Malays), 22.4% Chinese, 6.5% Indians, and 0.8% other ethnicities [19]. This diverse ethnic composition has cultivated a rich food culture, with each group contributing distinct traditional dishes, preparation methods, and portion norms. Such variety presents unique challenges for dietary assessment, as tools must capture ethnically specific foods, mixed dishes, regional terminology, and culturally relevant portion sizes to ensure accuracy [14,20]. To address this, we describe the development and validation protocol for the first version of Intake24 in the 3 main languages spoken in Malaysia—Bahasa Melayu, Mandarin, and Tamil—to ensure inclusivity and applicability across Malaysia’s major ethnic groups. This study aims to develop and validate Intake24 Malaysia (Intake24-MY) as a digital dietary assessment tool for the Malaysian population, by localizing it across Bahasa Melayu, Mandarin, and Tamil and adapting the food list, portion-size images, associated food prompts, and food composition database to reflect Malaysia’s multicultural dietary context.

In addition to tool development, this study will assess the usability and acceptability of Intake24-MY through small-scale and pilot testing. It will also evaluate its agreement with observed intake and interviewer-led 24hDRs. We hypothesize that Intake24-MY will be acceptable and usable among Malaysian adults and that dietary intake estimates collected using Intake24-MY will show acceptable agreement with reference dietary assessment methods. This system integrates the 3 main languages in Malaysia, that is, Bahasa Melayu, Mandarin, and Tamil. This involved the addition of foods to the database to cover the diverse range of foods and dishes consumed in the region, the inclusion of food photographs for portion-size estimation of regional foods, and linkage to food composition data. Once developed, the Intake24-MY system can serve as a platform for low-cost, large-scale collection of detailed dietary data. It may also serve as an exemplar for the development of Intake24 for other ASEAN countries.

## Methods

### Study Design

**Figure 1.**
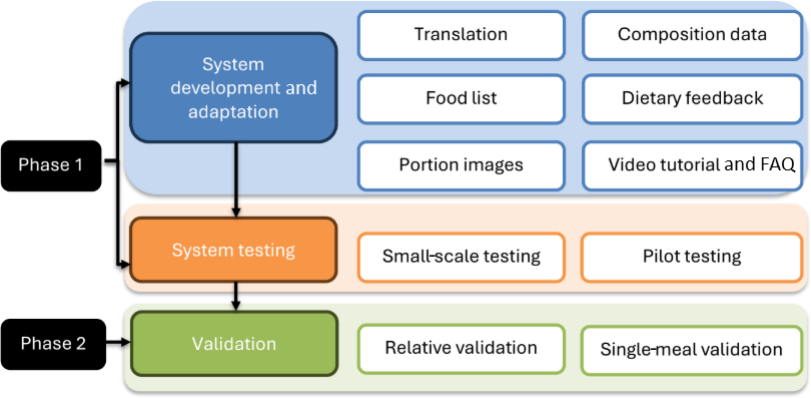
Two phases of Intake24-MY. FAQ: frequently asked questions.

The localization and implementation of Intake24 for Malaysia will be conducted in 2 phases, as illustrated in Figure 1. Phase 1 will focus on the development, cultural adaptation, photograph validation, and pilot testing of Intake24-MY, whereas phase 2 will focus on validation of the adapted system. All studies will be conducted as cross-sectional studies.

To ensure cultural and linguistic inclusivity, Intake24-MY will be localized to reflect Malaysia’s main languages and food culture by adapting the food list, portion-size estimation methods, associated food prompts, food composition database, and user support materials. The key features of Intake24-MY and their relevance in a Malaysian multicultural setting are summarized in Table 2.

**Table 2.** Key features of Intake24-MY and their relevance in a Malaysian multicultural setting.

Feature	Purpose	Benefit in the Malaysian context
User-end features		
Searchable multilingual food database	Enables users to find foods quickly by typing names in Bahasa Melayu, English, or Tamil	Captures ethnic-specific foods and local terminology across Malaysia’s multiethnic population
Culturally adapted portion-size images	Provides visual aids for estimating portion sizes	Improves accuracy for diverse dishes (eg, *nasi lemak*, *roti canai*, *laksa*), considering local serving styles
Multiple-pass recall prompts	Guides respondents through a structured set of steps (quick list, detail prompts, forgotten foods, portion sizes, review)	Minimizes omissions and improves completeness of recall data
Detailed food descriptors	Allows specification of brand, preparation method, and ingredients	Accounts for nutrient differences between traditional recipes and commercial products
Researcher-end features		
Automatic portion-to-weight conversion	Links portion images to gram weights in the food composition database	Ensures consistent, standardized nutrient calculation
Customizable food list	Enables inclusion of country-specific foods and beverages	Reflects Malaysia’s diverse dietary patterns, including festive and seasonal foods
Integration with the national food composition databases	Connects food items to nutrient values from available databases	Improves relevance and accuracy of nutrient estimates for the Malaysian population
Exportable datasets	Allows the data to be downloaded for analysis	Facilitates efficient statistical analysis and reporting for research purposes

This study protocol was developed in accordance with the STROBE-nut (Strengthening the Reporting of Observational Studies in Epidemiology—Nutritional Epidemiology) guidelines [19], with a checklist as presented in Checklist 1. The final study findings will adhere to the set standard to ensure comprehensive and transparent reporting.

### Study Setting

This study will be conducted in Peninsular Malaysia, which is home to approximately 83.4% of Malaysians [20]. The photograph validation, pilot study, and single-meal validation study will be conducted in the Central region. At the same time, the relative validation will be carried out across the East Coast, Northern, Southern, and Central regions to capture a wide range of dietary habits and sociodemographic diversity.

### Study Participants

The photograph validation has been completed among 24 participants. Both the pilot study and the single-meal validation study will recruit 100 participants each. The pilot study participants will be distributed as follows: 50 for the Bahasa Melayu version, 40 for the Mandarin version, and 10 for the Tamil version, reflecting diverse age groups, sex distributions, ethnicities, and socioeconomic backgrounds. The single-meal validation study will be conducted only for the Bahasa Melayu version. The relative validation study will recruit at least 482 participants. To ensure adequate testing of each language version of the system, participants will be stratified by language based on the Malaysian ethnic population distribution [20]. Approximately 71.1% (343 participants) will complete the Bahasa Melayu version, 22.4% (108 participants) will complete the Mandarin version, and the remaining 6.5% (31 participants) will complete the Tamil version.

### Eligibility Criteria

The eligibility criteria for all studies are that participants must be Malaysian adults aged 18 to 59 years. Additional criteria for the pilot and relative validation studies are that participants must be able to read, write, and communicate in Bahasa Melayu, Mandarin, or Tamil, and that they must have access to an internet-connected device. The photograph validation and single-meal validation studies require participants who can read and write in both English and Bahasa Melayu. Individuals will be excluded if they are pregnant, regularly fast, or follow a special diet that significantly restricts food or nutrient intake.

### Recruitment Process

Participants will be recruited using snowball sampling with trained data collectors for each study phase. Recruitment strategies will include social media outreach, poster distribution, institutional networks, and referrals through personal and professional contacts. Interested individuals will receive an explanatory statement for the study and will be required to complete an online expression-of-interest form and provide an informed consent form. The recruitment and data collection period are expected to span between 6 months and 1 year.

### Phase 1: Development and Cultural Adaptation of Intake24-MY

#### System Translation

The first step in the development process involves the translation of sections of the system that participants interact with into Malaysia’s 3 major languages: Bahasa Melayu (the national language) [21], Mandarin (the most widely spoken Chinese dialect in Malaysia) [22], and Tamil (commonly used within the Tamil community) [23]. Machine translation tools, such as Google Translate and Bing Translate, as well as AI-based assistance (eg, ChatGPT), will be used to provide initial drafts that bilingual translators will further refine. Subsequently, a team of bilingual reviewers will collaborate with the translators to further improve readability, consistency, and contextual relevance.

#### Food Lists

The development of the Intake24-MY food list process will commence with a review of the Australian Intake24 food list, which comprises 2226 items. Globally familiar items, such as ice cream, bread, and eggs, will be retained, whereas nonrelevant items will be removed. The retained food and drink items will be updated with relevant food composition data to reflect local values. Subsequently, 680 prepared or cooked foods and drinks from the Malaysian Food Composition Database (MyFCD) [24] will be incorporated. To address remaining gaps, additional food items will be identified through expert knowledge, popular restaurant menus, food blogs, food delivery apps, and major grocery outlets.

#### Portion-Size Images, Standard Portions, and Photograph Validation

**Figure 2.**
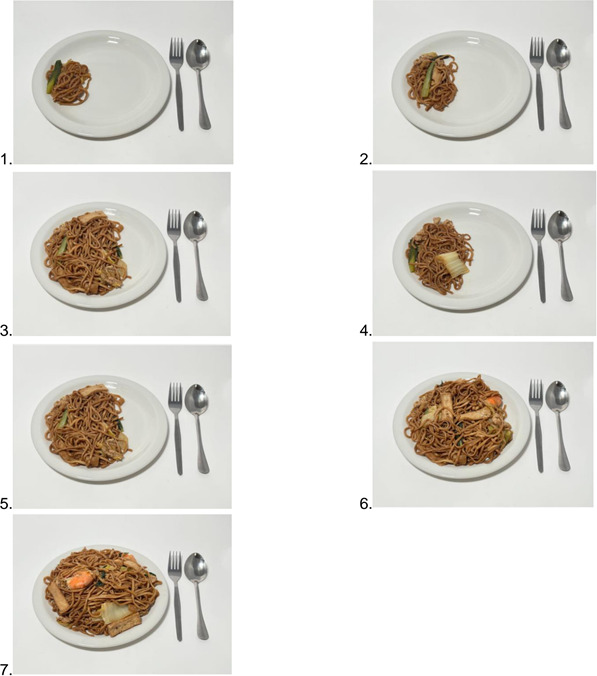
Example of as-served images for fried noodles. The portion size gradually increases across 7 photo series (1-7).

**Figure 3.**
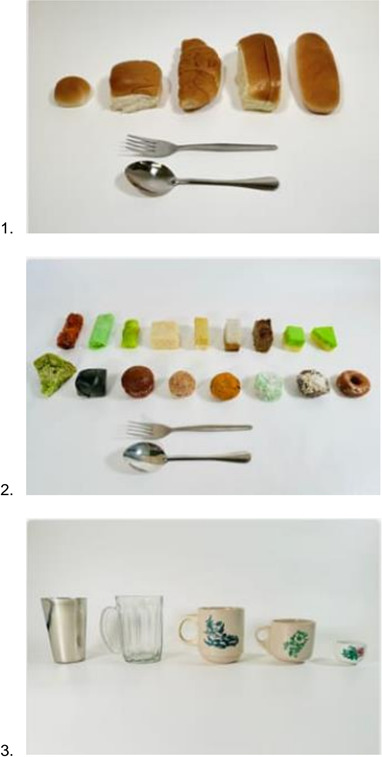
Examples of guide images: (1) cream buns, (2) Malaysian *kuih*, and (3) hot-drink mugs.

Intake24 offers 4 portion-size estimation options: as-served images, guide images, a sliding scale for drinks, and standard portion descriptions [25]. As-served images show a series of 7 photographs depicting incremental food portions, as shown in Figure 2. In contrast, guide images illustrate size differences for fixed items, such as cream buns, Malaysian *kuih,* and hot-drink mugs, as shown in Figure 3.

A total of 527 images and 160 standard portions from the Australian Intake24 database will be retained for use in Malaysia, particularly for common items such as packaged snacks and beverages, as well as for items that use similar drinkware and tableware. Where applicable, one-to-one visual matches, such as rice on a plate, ice cream in a bowl, and peanut butter spread on bread, will also be reused. To represent local foods not found in the Australian database, 210 new image sets, comprising as-served and guide images, will be created using an iPhone 15 Pro, a light box, and standard-size crockery, following standard guidelines [26].

A subset of newly developed food photographs will be selected for validation. The selected foods will represent a range of appearances, textures, consistencies, and portion characteristics, including beverages, liquid foods, mixed dishes, solid foods with undefined portion sizes, foods served as distinct pieces, single-item foods, preportioned foods, and foods commonly eaten in combination or as part of a meal [27].

A small-scale validation of the food photographs will be conducted using the “food-in-front” technique. Participants will be asked to fill out a form to estimate the portion sizes of foods placed in front of them using the corresponding portion-size images (Multimedia Appendix 1). Their estimated weights will then be compared with the actual measured weights of the foods [28]. Based on the results, a decision will be made on whether to include the image series in Intake24-MY or to use a standard portion option instead.

#### Compositional Data and Recipe Calculation

The food lists will be linked to the appropriate food composition tables. As MyFCD mainly includes generic foods and a limited number of prepared dishes [24], additional data will be sourced from databases in Singapore, Indonesia, and other countries, prioritized by relevance [29].

MyFCD provides macronutrient data for most foods, but micronutrient information is limited. Where no suitable match exists, recipe calculations will be used to estimate nutrient content. Food matching will follow the Food and Agriculture Organization guidelines [30], with quality codes and source references recorded. For unmatched dishes, detailed recipes with ingredient weights and cooking methods will be used, applying nutrient retention and yield factors [30,31]. These data will be compiled into a new food composition database.

#### Dietary Feedback

The feedback component will be adapted to reflect the Malaysian Dietary Guidelines [32] and will be provided to users upon completion of their dietary recalls. The feedback will be age-specific and sex-specific and will provide culturally relevant guidance on potential dietary adjustments to promote healthier eating patterns.

#### Video Tutorial and Frequently Asked Questions

A screen-capture walkthrough will be developed to guide users through the Intake24-MY system. It will demonstrate various tasks within the system, such as searching for food items, matching foods within the database, reporting portion sizes, using the “same as before” option, prompting for associated foods, and submitting dietary recalls. A voice-over explaining the process will be recorded in each of the 3 languages: Bahasa Melayu, Mandarin, and Tamil. Additionally, frequently asked questions (FAQ) documents in all 3 languages will also be prepared, featuring screenshots to support user navigation.

#### Small-Scale and Pilot Testing

A small-scale system test will be conducted first to identify any missing food items, technical issues, and user challenges. This will be followed by pilot testing, where participants will complete 2 nonconsecutive weekday dietary recalls using Intake24-MY and an interviewer-led 24hDR. The participants will be asked to provide feedback on language, missing food items, and system usability. System refinements will then be implemented by developers, prioritizing critical issues based on their impact and severity.

#### System Refinement

**Figure 4.**
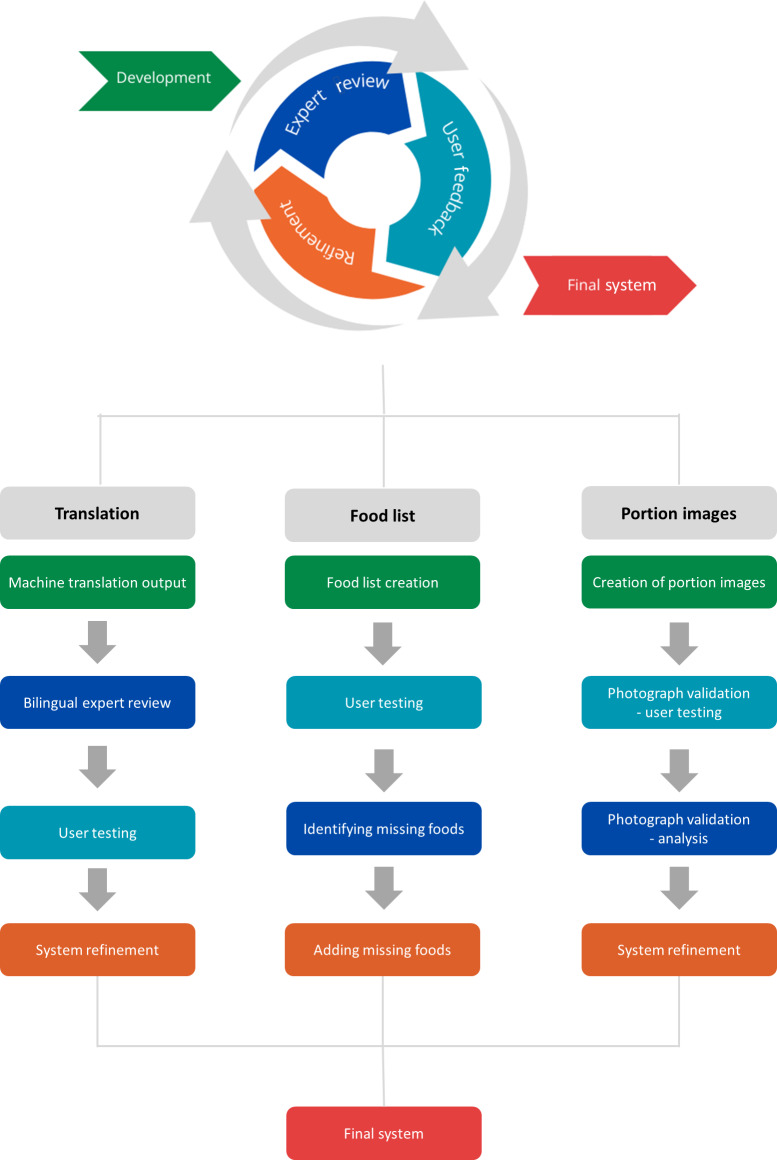
Intake24-MY system refinement iterative design process.

Intake24-MY will adopt a user-centered, iterative approach to system localization, consistent with previous adaptations [33]. Each development stage will be followed by expert review, user testing, and system refinement, as shown in Figure 4.

Different methods will be applied at each stage of the iterative process. Translation will begin with machine-generated drafts, which will be refined by bilingual experts and adjusted based on user feedback. The food list will first be compiled from existing data sources, and then expanded to include any missing or culturally specific items. Portion-size images will undergo photograph validation to assess their appropriateness. The iterative cycle will continue through small-scale testing, pilot testing, and relative validation phases.

### Phase 2: Validation Study

#### Single-Meal Validation

Participants will be asked to estimate their meal intake using Intake24-MY and compare it with direct observation. On the first day, they will be served a single meal and asked to consume it while leaving any leftovers at the study site. The next day, participants will receive an email with a link to log into Intake24-MY to complete a recall of the previous day’s lunch. They will be asked to identify the food and drink items and their corresponding portion sizes. Trained researchers will observe the participants, weigh the foods served, and measure any leftovers.

Data collected using Intake24-MY will be downloaded and compared with the food and drink items and portion sizes recorded by the observer for each individual. Foods will be coded as matches, omissions, or intrusions. When a match is found, the accuracy of the portion-size estimates will be assessed by calculating the ratio of the food weight reported in Intake24-MY to the weight measured by the observer.

#### Relative Validation (Data Collection)

Participants will first provide demographic information and then complete a dietary recall using Intake24-MY and an interviewer-led 24hDR. The interviewer-led 24hDR will be conducted using the externally validated multiple-pass method [34] and will be used as the reference method for comparison with Intake24-MY.

For the pilot study and relative validation, the dietary data will be collected over 2 nonconsecutive days and 4 nonconsecutive days, including 1 weekday and 1 weekend day, across 2 and 3 weeks, respectively. On each assessment day, dietary intake during the previous 24 hours will be assessed using both methods with the same reference day. Participants will receive a unique link by email or text message to access the Intake24-MY system. They will follow the system instructions to complete the dietary recalls. For the interviewer-led dietary recall, the first session will be conducted face-to-face, with participants completing dietary recall under the interviewer’s guidance. Subsequent dietary recall interviews will be conducted by phone. To minimize potential order effects [33], participants will be randomly assigned as a counterbalance measure: 75% will begin with Intake24-MY, followed by the interviewer-led recall to evaluate sequential performance, while 25% will start with the interviewer-led recall, followed by Intake24-MY. Once they finish their final recall, participants will be asked to give feedback (Multimedia Appendix 2) and assess usability through a structured questionnaire (Multimedia Appendix 3).

Dietary data collected from the interviewer-led 24hDRs will be analyzed using Nutritionist Pro software (Axxya Systems). Reported foods and beverages will be coded and matched to food items available in the software database, supplemented with MyFCD values, regional food composition databases, and recipe calculations where required. Portion sizes reported during the interviews will be converted to grams before nutrient analysis. For mixed dishes, ingredients and serving sizes will be estimated based on participant-reported recipes, standard recipes, or commonly used preparation methods.

#### Interviewer Training and Standardization

A multiple-pass protocol, developed based on the USDA Automated Multiple-Pass Method [34] and adapted to the Malaysian context, will be used together with a 24hDR form (Multimedia Appendix 4). The interviewer-led 24hDR will follow a standardized 5-pass approach.

Participants will first provide a quick list of all foods and beverages consumed on the previous day, followed by prompts to identify any forgotten items. Contextual information, including the time of intake, eating location, and source of food, will then be collected. A detailed pass will gather information on food descriptions, cooking methods, brand names, ingredients, and serving sizes for mixed dishes, as well as portion-size estimates, using food photographs, flipcharts, and standard household measures. Finally, the interviewer will review the recall with the participant and probe for any additional items before closing the session.

**Figure 5.**
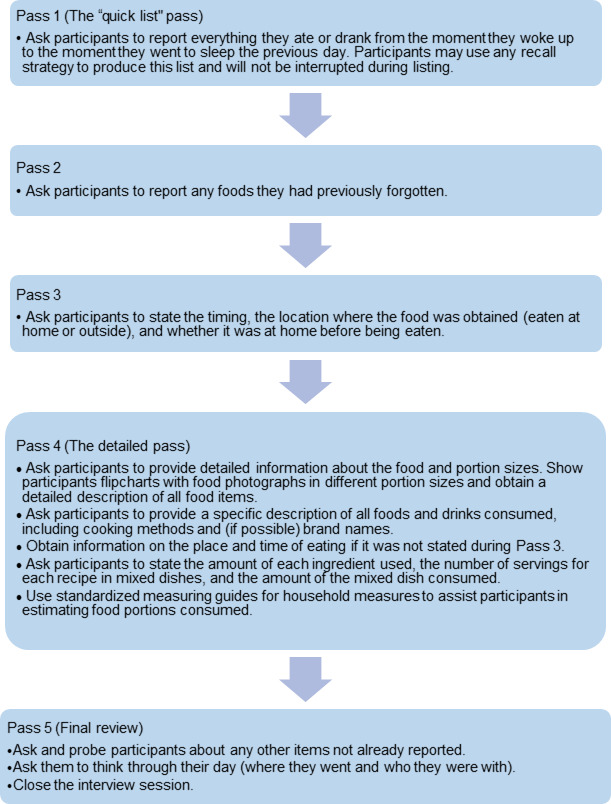
Multiple-pass method for interviewer-led 24-hour dietary recall.

**Figure 6.**
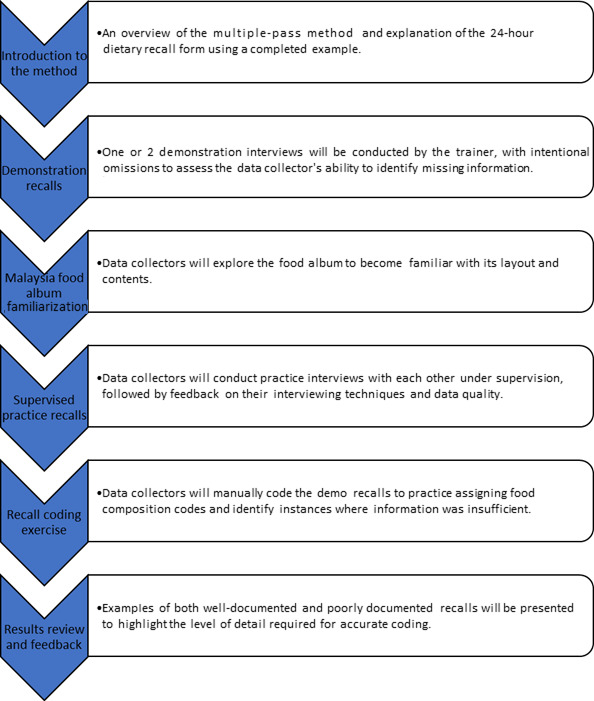
The training procedure for data collection.

These structured procedures, including forgotten-food probes, portion-size estimation aids, interviewer training, and a final review, are intended to minimize recall bias, misreporting, and interviewer-related variation. Data collectors will receive comprehensive training to ensure the consistent and accurate administration of interviewer-led dietary recalls. The Malaysia Food Album [35] will be used to aid portion-size estimation. A complete interview guide is shown in Figure 5, and the training procedure is shown in Figure 6.

### Statistical Methods

The impact of errors in reported nutrient intakes will be investigated by calculating the ratio of an individual’s energy and nutrient intakes from that meal, based on the Intake24-MY recall, to the energy and nutrient intakes measured by the observer.

A modified Bland-Altman method will be used to assess the agreement between Intake24-MY and interviewer-led 24hDRs, with a focus on accuracy and precision. Given the potential nonnormality of dietary intake data, nutrient values will be log-transformed before analysis. To evaluate the effect of recall order, the ratios of geometric means and SDs will be compared between participants who begin with Intake24-MY and those who start with the interviewer-led dietary recall.

### Ethical Considerations

This study has received ethical approval from the Monash University Human Research Ethics Committee (MUHREC ID 41337). Informed consent will be obtained from all participants before enrollment. The study poses minimal risk, and participation is voluntary. Individuals can withdraw from the study at any time without penalty. All data will be treated confidentially, stored securely in password-protected accounts, and used solely for research purposes.

## Results

This study was funded in December 2023, with data collection commencing in June 2024 and currently ongoing. A total of 24 participants completed the photograph validation study, while 40 participants were recruited for the pilot study, and 74 participants joined the single-meal validation study. Data analysis is anticipated to begin in August 2026, with results expected to be published in 2027.

## Discussion

### Overview

This paper describes the protocol for a study designed to determine whether the Intake24-MY system is comparable to interviewer-led 24hDR dietary assessment among the Malaysian population. This adaptation protocol will be the first multicultural and multilingual adaptation of the Intake24 system in Southeast Asia. This study describes an adaptation process that relies heavily on an iterative user-testing and refinement approach.

As a LMIC, Malaysia would benefit from an efficient approach to adapting existing systems rather than developing new ones from scratch. Adaptation can build on established online dietary assessment tools that have demonstrated effectiveness in capturing dietary intake. This reduces time and cost while avoiding duplicative efforts, allowing for more focus on addressing the implications of implementing a system suitable for a multicultural population.

### Comparison With Prior Work

Although adaptation offers clear practical advantages, it also presents challenges. Most existing systems, such as the Automated Self-Administered 24-hour dietary assessment tool (ASA24) (United States, Canada, and Australia) [36], myfood24 [37], Intake24 (United Kingdom) [17], R24W (France, Canada) [38], and GloboDiet (Europe) [39], are grounded in Western dietary patterns. As a result, they often lack adequate representation of staple foods, complex mixed dishes, and culturally specific preparation methods common in South Asian diets, such as the wide variety of rice, noodles, curry dishes, fermented foods, and shared accompaniments. This highlights the need for careful cultural and contextual validation to ensure their relevance and usability within the Malaysian context.

Among the tools mentioned above, Intake24 stands out for its open-source model and flexibility, making it more adaptable to cultural contexts than more restrictive or proprietary systems. Intake24 also demonstrates a strong user-centered design, which contributes to consistently high usability. In its initial development with Scottish participants aged 11 years and older, 83% found the system easy to follow and understand, and 78% felt it accurately captured their intake [40,41]. Following adaptation in New Zealand, usability remained high, with 95% and 84% of users reporting the same outcomes, respectively [42]. Intake24 has also been successfully localized for South Asia [43], offering a strong foundation for further adaptation to Malaysia’s unique multicultural context.

The adaptation process will involve substantial changes to the food list, which will be expanded to include commonly prepared and cooked items from Malaysia’s major ethnic cuisines. A new food coding system will be developed using MyFCD as a foundation, but restructured to include custom food groups and location-specific codes to support traceability, effective grouping, and long-term system maintenance. Food names will be tailored to reflect regional variations and colloquial terms, ensuring both clarity and cultural relevance. Associated food prompts will be redesigned to better reflect typical Malaysian meals. This includes more open-ended questions and targeted follow-ups for multicomponent dishes, allowing the system to capture key elements such as sauces, condiments, and shared side dishes. Portion-size estimation will also be adapted using new photographs of Malaysian foods. Food composition matching and recipe calculations will be conducted in accordance with Food and Agriculture Organization (FAO) guidelines [30,31]. Finally, the entire user experience will be made available in Bahasa Melayu, Mandarin, and Tamil to ensure accessibility across Malaysia’s main language groups.

### Limitations

The validation of Intake24-MY will be limited to adults residing in Peninsular Malaysia and may not be generalizable to other age groups or regions, particularly East Malaysia. While Intake24-UK has been validated across a wider age range in the United Kingdom [17], further adaptation and validation will be needed for Malaysian children and older adults, especially those with limited digital literacy. In addition, East Malaysia, located on the island of Borneo, is home to many of the country’s Indigenous populations [19] and differs from Peninsular Malaysia in terms of language, food preferences, food terminology, and portion-size norms. Targeted cultural and linguistic adaptation will therefore be required before Intake24-MY can be extended to this region.

The sample allocation for the relative validation study is based on Malaysia’s population distribution rather than on equal numbers across the 3 language versions. While this approach supports the assessment of overall agreement in the intended target population, language-specific findings will be interpreted descriptively.

Nevertheless, this study may provide a useful foundation for extending Intake24-MY to other demographic groups and geographic regions, subject to further cultural adaptation and validation. It may also offer valuable insights into the process of multicultural adaptation, both within Intake24 and in the development of other dietary assessment tools.

### Conclusions

This protocol describes the cultural and linguistic adaptation of Intake24 for Malaysia and the planned validation of Intake24-MY against interviewer-led 24hDRs. The study will evaluate whether Intake24-MY can provide comparable dietary intake estimates and acceptable usability among Malaysian adults. If validated, the system may offer a scalable digital option for dietary assessment in Malaysia’s multicultural population and provide a framework for future adaptations to other population groups and Southeast Asian settings.

## Supplementary material

10.2196/90106 Multimedia Appendix 1 Photograph validation form.

10.2196/90106 Multimedia Appendix 2 Feedback form.

10.2196/90106 Multimedia Appendix 3 System Usability Scale questionnaire.

10.2196/90106 Multimedia Appendix 4 24-Hour dietary recall form.

10.2196/90106 Checklist 1 STROBE-nut: an extension of the STROBE statement for nutritional epidemiology.

## Abbreviation

24hDR: 24-hour dietary recall
FAO: Food and Agriculture Organization
FAQ: frequently asked questions
Intake24-MY: Intake24 Malaysia
LMIC: low- and middle-income country
MyFCD: Malaysian Food Composition Database
STROBE: Strengthening the Reporting of Observational Studies in Epidemiology—Nutritional Epidemiology
