# Supplementary material for: A Digital Dietary Assessment Tool for a Multicultural Population (Intake24 Malaysia): Protocol for a Development and Validation Study
Source: JMIR Res Protoc. 2026 Aug 4;15:e90106. doi: 10.2196/90106 (PMC13436792; doi:10.2196/90106)
Supplement: Supplementary file 1 [file resprot-v15-e90106-s001.pdf]

CODE:

MONASH University MALAYSIA

Recording sheet

Instructions: Please answer all the questions below. Columns coloured in grey should be left blank.
Arahan: Sila jawab semua soalan di bawah. Sila biarkan kosong bagi kolom berwarna kelabu.

No.	Food	Guide images		As-served images														
		Picture number	Number	<	1		2		3		4		5		6		7	<
**Station 1**																		
1.	Sambal				/	> 1/2												
2.	White rice/ Nasi putih																	
3.	Malaysian kuih/Kuih Malaysia	1	1/2															
4.	Muffins & cupcakes/ Muffin & kek cawan																	
5.	Instant noodles/ Mi segera																	
6.	3-in-1 sachet/ Minuman 3 dalam 1																	
7.	Chicken soto/ Soto ayam																	

CODE:

MONASH University MALAYSIA

No.	Food	Guide images		As-served images														
		Picture number	Number	<	1		2		3		4		5		6		7	<
8.	Lontong darat																	
9.	Kaya bread/ Roti kaya																	
**Station 2**																		
10.	Iced tea/ Teh ais																	
11.	Iced Boba tea/ Air teh Boba																	
12.	Asian sweet porridge/ Bubur manis																	
13.	Bistik egg/ Telur bistik																	
14.	Keropok lekor																	
15.	Guava/ Jambu batu																	
16.	Varuval																	
17.	Pulut kuning																	
18.	Asian salad/ Kerabu																	

CODE:

No.	Food	Guide images		As-served images														
		Picture number	Number	<	1		2		3		4		5		6		7	<
**Station 3**																		
19.	Asian dessert/ Pemanis mulut																	
20.	Porridge/ Bubur																	
21.	Curry/Kari																	
22.	Kuey teow gravy/Kuey teow basah																	
23.	Paneer gravy/Paneer berkuah																	
24.	Noodles/Mi																	
25.	Egg/Telur																	
26.	Malaysian biscuit/ Biskut Malaysia																	
27.	Chilli pan mee/Pan mi cili																	

CODE:

MONASH University MALAYSIA

Comments/Notes:

Checked by:

Name:

Date:
