# Supplementary material for: A Digital Dietary Assessment Tool for a Multicultural Population (Intake24 Malaysia): Protocol for a Development and Validation Study
Source: JMIR Res Protoc. 2026 Aug 4;15:e90106. doi: 10.2196/90106 (PMC13436792; doi:10.2196/90106)
Supplement: Supplementary file 2 [file resprot-v15-e90106-s002.pdf]

CODE

FEEDBACK QUESTIONNAIRE

1. I would like to use Intake24 often
- Disagree strongly
- Disagree
- Neither agree nor disagree
- Agree
- Agree strongly

2. I would need help using Intake24 -
- Disagree strongly
- Disagree
- Neither agree nor disagree
- Agree
- Agree strongly

3. Intake24 was easy to use.
- Disagree strongly
- Disagree
- Neither agree nor disagree
- Agree
- Agree strongly

4. Intake24 was too complicated.
- Disagree strongly
- Disagree
- Neither agree nor disagree
- Agree
- Agree strongly

5. The different parts of Intake24 worked well together.
- Disagree strongly
- Disagree
- Neither agree nor disagree
- Agree
- Agree strongly

6. Intake24 was too inconsistent.
- Disagree strongly
- Disagree
- Neither agree nor disagree
- Agree
- Agree strongly

7. Most people would learn to use Intake24 quickly.

- Disagree strongly
- Disagree
- Neither agree nor disagree
- Agree
- Agree strongly

8. Intake24 is awkward to use.

- Agree strongly
- Agree
- Neither agree nor disagree
- Disagree
- Disagree strongly

9. I felt very confident using Intake24.

- Agree strongly
- Agree
- Neither agree nor disagree
- Disagree
- Disagree strongly

10. I needed to learn a lot before I could use Intake24.

- Agree strongly
- Agree
- Neither agree nor disagree
- Disagree
- Disagree strongly

11. Intake24 captured my diet accurately.

- Agree strongly
- Agree
- Neither agree nor disagree
- Disagree
- Disagree strongly

12. I like how Intake24 looks.

- Agree strongly
- Agree
- Neither agree nor disagree
- Disagree
- Disagree strongly

13. I felt I was able to complete Intake24 in a reasonable amount of time.

- Agree strongly
- Agree
- Neither agree nor disagree
- Disagree

CODE

- Disagree strongly

14. I will make changes to my diet as a result of completing Intake 24.
- Agree strongly
- Agree
- Neither agree nor disagree
- Disagree
- Disagree strongly

15. Would you have preferred to give information about your diet via Intake24, or via answering questions from the interviewer?
- Intake24
- From the interviewer
- No preference
- Don’t know

16. Do you feel you gave the most accurate information about your diet when using Intake24 or through the questions you answered from the interviewer?
- Through Intake24
- From the interviewer
- No difference
- Don’t know

17. If you chose to receive feedback on your diet in Intake24, what did you think of it?
- Very useful
- Somewhat useful
- Not very useful
- Not useful at all
- I did not receive feedback on my diet

18. Did you have any problems logging in to Intake24?
- Yes
- No
- Don’t know

19. Did you have any problems with finding different types of foods in Intake24?
- Yes
- No
- Don’t know

Details

20. Did you have any problems with the portion size selection in Intake24?
- Yes
- No
- Don’t know

21. What problems did you have with the portion size selection in Intake24?

Details

22. Did you have any problems when entering missing food items in Intake24?
    - Yes
    - No
    - I did not enter any missing food items
    - Don’t know

23. What problems did you have when entering missing food items in Intake24?

Details

24. Was it difficult or easy for you to understand the language used in the system?
- Very difficult
- Difficult
- Not difficult, Not that easy
- Easy
- Very easy

25. If it was difficult, please indicate which part of the system. (categories portion size)

CODE

26. What changes would you like us to make to rectify the language used?

27. Do you have any other comments from your experience using Intake24?

Many thanks for taking part in this study – we really appreciate your feedback about Intake24.
