# Supplementary material for: A Digital Dietary Assessment Tool for a Multicultural Population (Intake24 Malaysia): Protocol for a Development and Validation Study
Source: JMIR Res Protoc. 2026 Aug 4;15:e90106. doi: 10.2196/90106 (PMC13436792; doi:10.2196/90106)
Supplement: Supplementary file 3 [file resprot-v15-e90106-s003.pdf]

System Usability Scale (SUS) Questionnaire

Instruction: For the following statements below, please tick (/) the most appropriate answer based on the scale provided.

1. Strongly disagree
2. Disagree
3. Neutral
4. Agree
5. Strongly agree

No.		1	2	3	4	5
1.	I think that I would like to use this system frequently					
2.	I found the system unnecessarily complex					
3.	I thought the system was easy to use					
4.	I think that I would need the support of a technical person to be able to use this system					
5.	I found the various functions in this system were well integrated					
6.	I thought there was too much inconsistency in this system					
7.	I would imagine that most people would learn to use this system very quickly					
8.	I found the system very cumbersome to use					
9.	I felt very confident using the system					
10.	I needed to learn a lot of things before I could get going with this system					
