# Supplementary material for: A Digital Dietary Assessment Tool for a Multicultural Population (Intake24 Malaysia): Protocol for a Development and Validation Study
Source: JMIR Res Protoc. 2026 Aug 4;15:e90106. doi: 10.2196/90106 (PMC13436792; doi:10.2196/90106)
Supplement: Supplementary file 4 [file resprot-v15-e90106-s004.pdf]

24H Diet Recall Form

Date of the interview:			Interviewer's name:			Subject's code:	
Recall day (Circle the recall day)	Monday	Tuesday	Wednesday	Thursday	Friday	Saturday	Sunday
Meal and beverage	Time	Location (Home / outside)	Meal and beverage description			Amount/portion size	Meal weight (g/ml)
			Cooking method	Ingredients /recipe	Brands/franchise		
